# Masculine depression and acute mental health burden

**DOI:** 10.1038/s41598-026-44727-7

**Published:** 2026-04-05

**Authors:** Claudia von Zimmermann, Christian Weinland, Johannes Kornhuber, Bernd Lenz, Christiane Mühle

**Affiliations:** 1 Department of Psychiatry and Psychotherapy, Friedrich-Alexander-University Erlangen-Nürnberg (FAU), Schwabachanlage 6, 91054 Erlangen, Germany; 2 Department of Addictive Behavior and Addiction Medicine, Central Institute of Mental Health (CIMH), Medical Faculty Mannheim, Heidelberg University, Mannheim, Germany

**Keywords:** Male depression, Masculine depression, Mental health burden, Anxiety, Help-seeking, Diseases, Health care, Psychology, Psychology

## Abstract

Masculine depression is characterized by externalizing symptoms, including anger, aggression, substance use, and risk-seeking behavior. However, the understanding of its clinical presentation remains highly limited. Here, we tested mental health burden in patients with high masculine depression scores in a case-control study of 163 depressed in-patients (44% women and 56% men; high vs. low masculine depression scores according to a sex-separated median split of the Male Depression Risk Scale-22 [MDRS-22]) and 176 controls (51% women and 49% men). In models adjusted for depression severity, the group of patients with a high masculine depression score (compared to those with a low score) was related to a greater acute mental health burden as measured by the Symptom Checklist-90-Revised (SCL-90-R). This association was evident across the three global indices of the SCL-90-R. Moreover, the dimensions somatization, anger-hostility, paranoid ideation, and psychoticism were associated with the group of patients with high masculine depression scores relative to the group of patients with low masculine depression scores as well as to higher MDRS-22 scores. Here, we provide evidence that patients with high masculine depression scores experience a substantial mental health burden – regardless of their biological sex. This is important, as patients with masculine depression were shown to be undertreated.

**Trial registration** German Clinical Trials Register ID DRKS00015291, Registration Date 2020-01-06.

**Supplementary Information:**

The online version contains supplementary material available at 10.1038/s41598-026-44727-7.

## Introduction

The concept of Masculine Depression represents a relativeley novel subtype which focuses on atypical depressive symptoms. It was initially described in the 1990s as a form of depression in men characterized by a lower frequency of typical depressive symptoms^1^. Historically, depression has been regarded as a predominantly “female” disorder with prevalence rates reported to be approximately twice as high in women compared to men^2^. However, the use of more specific screening tools that also capture externalizing symptoms has revealed comparable prevalence rates of depression in both sexes^3^. These more externalizing symptoms encompass anger, aggression, distraction, avoidance, emotional suppression, irritability, substance use, and risk-seeking behavior^4,5^. Additionally, an increased risk of suicidality is also assumed to be associated with masculine depression^6^.

To better identify such symptom profiles of the so-called masculine depression, screening tools have been developed, including the Gotland Male Depression Scale (GMDS)^7^, the Male Depression Risk Scale 22 (MDRS-22)^4^, and the Gender-Sensitive Depression Screening (GSDS)^8,9^. Importantly, when applied to women, these instruments have shown that masculine depression can also occur in females, particularly in the context of stress exposure^10^. Hence, the term “masculine depression” is used in this study to describe a behavioral profile rather than a sex-specific disorder. Nevertheless, this terminology should be interpreted cautiously, as it risks attributing behavior perceived as maladaptive or socially undesirable to certain gendered groups.

Patients with more pronounced symptoms of masculine depression show stronger borderline, impulsive, and antisocial personality accentuations than those with lower symptoms of masculine depression^11^. Those cluster B personality disorders are characterized by impulsive, dramatic, emotionally unstable, and erratic behaviors. Especially the borderline personality disorder often co-occurs with an altered stress response^12^, suggesting that stress regulation may be a central component linking externalizing symptoms and behavioral dysregulation. Extending this framework, previous work from our cohort has shown that higher masculine depression scores are associated with longer working hours^13^. Importantly, prolonged working hours are linked to adverse mental and physical health outcomes^14–16^, as well as increased alcohol and substance use in both men and women^39^. These patterns suggest that traditional masculine norms may shape coping behaviors, such as overwork or substance use, which in turn may contribute to the expression of the masculine depression phenotype.

Beyond personality and behavioral correlates, several psychosocial and developmental factors may contribute to the emergence of masculine depression symptoms. Maladaptive early childhood schemas, as suggested by Chodkiewicz et al.^17^, particularly those related to feelings of disconnection and rejection as well as difficulties with autonomy and performance, and a strong emphasis on work^18^ may predispose individuals to develop externalizing depressive symptoms. Consistent with this, individuals with higher masculine depression scores tend to have fewer healthcare contacts and more frequent substance use, reflecting both under-treatment and maladaptive coping^13^. Within this framework, engagement in prolonged work hours can be seen not only as a coping strategy but also as a means of fulfilling traditional masculine norms. Engagement in work beyond standard hours has traditionally been more prevalent among men than women^19^, and women with children are more likely to leave male-dominated occupations compared with men or childless women^20^. With the increasing convergence of gender roles, such work-related behaviors and associated stress exposure may become more similar across men and women, potentially influencing the expression of externalizing depressive symptoms regardless of biological sex.

In summary, the masculine depression syndrome can tentatively be conceptualized as a stress- and behavior-related symptom constellation, in which sociocultural norms, coping strategies, and externalizing behaviors appear to interact and may influence both the expression and perceived severity of depressive symptoms, potentially contributing to externalizing manifestations of depression. Building on this conceptual model, the present study aimed to examine whether individuals with higher masculine depression scores exhibit greater acute mental health burden than those with lower scores. By focusing on mental health burden, rather than single symptoms alone, we sought to capture the broader impact of stress- and behaviorally-linked depressive symptomatology.

### Aims of the study

Here, we analyzed whether the mental health burden (measured using the Symptom Checklist-90-Revised (SCL-90-R)) differs between patients exhibiting more pronounced symptoms of masculine depression and those with fewer such symptoms (according to the MDRS-22), in order to more accurately characterize acute psychiatric symptomatology. Based on prior evidence linking masculine depression with both internalizing and externalizing symptoms, we hypothesized that: First, patients with higher masculine depression scores (HMD) will show greater overall mental health burden on the SCL-90-R compared to patients with lower scores (LMD), even after controlling for overall depression severity, age, and sex. Second, specific SCL-90-R symptom dimensions associated with externalizing behaviors, such as anger–hostility and aggression, will be elevated in HMD patients relative to LMD patients, reflecting a broader symptom profile beyond core depressive features. Third, both HMD and LMD patients will show higher symptom burden compared with healthy controls. Last, in an explorative approach, sex-specific analyses will examine whether the associations between HMD and SCL-90-R scores are independently present in male patients and female patients, testing the notion that masculine depression reflects a distinct symptom profile rather than being strictly sex-dependent. By focusing on behavioral and stress-related correlates of depression rather than assuming a distinct “male” subtype, this study seeks to clarify the clinical relevance of so-called masculine depression symptoms and its contribution to mental health burden.

## Methods

### Sample population

The study was conducted as a prospective, open-label, comparative cohort study with one single data collection point per participant as part of the Masculine Depression Project^11,13,21^. Recruitment of participants took place from May 2017 to November 2019.

We screened a total of 658 study subjects and included 170 patients (from the Department of Psychiatry and Psychotherapy at the Friedrich-Alexander-University Erlangen-Nürnberg (FAU) and the Clinic for Psychiatry, Addiction, Psychosomatics, and Psychotherapy at the Europakanal in Erlangen, Germany) and 176 healthy control subjects. Each visit lasted approximately 3–4 h and took place between 7 and 10 am. Inclusion requirements were: a minimum age of 18 years, a body mass index (BMI) between 18.5 kg/m^2^ and 35 kg/m^2^, and written informed consent. The BMI range was predefined to reduce metabolic and endocrine confounding in planned biomarker analyses. Healthy controls were recruited from the community using flyers and social networks. A telephone screening was conducted with those interested in the study.

Eligibility criteria for patients included an inpatient stay due to a moderate or severe depressive episode in one of the two clinics mentioned or depressive symptoms of a recurrent unipolar or bipolar affective disorder classified as moderate or severe according to International Classification of Disease (ICD)-10^22^ and Diagnostic and Statistical Manual of Mental Disorders (DSM)-5^23^. The criteria were systematically documented using a structured electronic checklist, with yes/no entries completed by a trained medical study staff together with the patient. Study recruitment had to take place during the first five days of hospitalization. Psychotic disorders led to exclusion from the study in the group of patients and in the healthy control group. Additional exclusion criteria for control subjects comprised regular intake of psychotropic drugs, a current psychiatric diagnosis according to ICD-10 (except for nicotine dependence), or a history of in-patient treatment. The control subjects received an expense allowance of 30 euros.

### Evaluation of the depression symptoms and current psychopathological symptomatology

The participants completed an extensive battery of computerized psychometric tests in our laboratory. Questionnaires were, in part, administered electronically via SoSciSurvey (www.soscisurvey.de). We used the MDRS-22^4^ to capture the characteristics of the masculine depression. It consists of 22 items to assess predominantly externalizing symptoms which are assumed to be typical for masculine depression and refers to the previous month. The scale includes six subscales that capture both internalizing symptoms (emotional suppression and somatic symptoms) and primarily externalizing symptoms (drug use, alcohol abuse, anger and aggression, and risk-taking). As no German version existed at the time of recruitment, our study team translated the version by Rice et al.^4^ into German using a forward–backward translation procedure involving three independent bilingual translators, followed by reconciliation and pilot testing within the study cohort (Supplementary Table S1). Each item is rated on a scale from 0 to 7, with higher values indicating more frequent symptom occurrence. Means were calculated across items, so the resulting MDRS-22 scores can range from 0 to 7^4^. Cronbach’s alpha was 0.828 in the patient group and 0.787 in the control group, indicating good internal consistency, comparable to a recently published German MDRS-22 version by Walther et al.^24^. For group-based analyses, participants were categorized using sex-specific median splits of MDRS-22 scores to allow within-sex comparisons while accounting for sex differences in score distributions. To improve comparability with other studies, a 7-point scale with an adapted median split was used in the present analysis. As a result, MDRS-22 scores differ from those reported in our previous work by Sedlinská et al.^11^. Males with a score at or above 1.70 and females with a score at or above 1.82 were classified as patients with HMD.

Psychological symptoms experienced during the past week were assessed using the Symptom Checklist-90-Revised (SCL-90-R)^25^. This instrument is widely used to assess symptom severity in acutely distressed individuals^24^. Participants rated all 90 items on a 5-point Likert scale ranging from ‘‘not at all’’ (0) to ‘‘extremely’’ (4). Cronbach’s alpha, calculated across all 90 SCL-90-R items, was 0.968 in the patient group and 0.930 in the control group, indicating good internal consistency. The SCL-90-R is a self-reported scale consisting of nine subscales: (1) somatization, (2) obsessive-compulsive, (3) interpersonal sensitivity, (4) depression, (5) anxiety, (6) anger-hostility, (7) phobic anxiety, (8) paranoid ideation, and (9) psychoticism. It includes three global indexes: (1) The Global Severity Index (GSI), an average score of all 90 items, reflecting one’s overall level of distress, (2) the Positive Symptom Total (PST) showing the number of symptoms affirmed, (3) the Positive Symptom Distress Index (PSDI) showing the intensity of the symptoms. We applied a normalization of the values in accordance with the manual of derogatis^25^ and show here normalized t-values with average values of 60 and maximum values of 80. Since the questionnaires were administered electronically, we tracked completion time to explore the hypothesis that participants with higher depressive symptom burden might perform the task more slowly or less efficiently, providing an additional behavioral measure of task engagement and cognitive functioning.

We used the Beck’s Depression Inventory-II (BDI-II)^26^ to determine depression severity. The self-report questionnaire consists of 21 items that evaluate the presence and intensity of depressive symptoms over the past two weeks.

### Statistical analyses

The depressed patients cohort was divided into two groups based on a sex-specific median split MDRS-22 scores: 81 patients with HMD and 82 patients with low masculine depression scores (LMD). Seven participants with missing MDRS-22 data were excluded from the analysis.

We used SPSS for Windows 29.0.1.0 (SPSS Inc., Chicago, IL, USA) to analyze the data and GraphPad Prism 8.4.3 (Graph Pad Software Inc., San Diego, CA, USA) as well as Microsoft Excel (Microsoft Corporation, Redmond, WA, USA) to visualize the results.

We tested variation in frequencies using χ^2^ tests (and we report P values from two-tailed Fisher’s exact test if at least one cell failed to reach an expected value of five observations). Correlations were analyzed using the Pearson method. We employed Student’s t-tests for differences in two independent groups, and the statistics were adjusted when necessary, according to the Levene’s test. We show medians and interquartile ranges (IQR) for SCL-90-R parameters. We computed the following regression models: Model 1: Binary logistic regression with patients with high versus low masculine depression (HMD vs. LMD) as dependent variable and with SCL-90-R scores as predictors. Sex, age, and BDI-II scores were included as covariates. Model 2: Linear regression analyses conducted within the depressed patient cohort only, with the continuous MDRS-22 score as the dependent variable and SCL-90-R scores as predictors. Sex, age, and BDI-II scores were included as covariates in all models. In addition, exploratory sex-separated subanalyses were performed without including sex as a covariate. Model 3: Binary logistic regression analyses with HMD patients vs. healthy control subjects (HCS) and LMD patients vs. healthy controls as dependent variables. Sex and age were included as covariates. The BDI-II score was used as covariate to assure that the observed effects are present after adjustment for depression severity in the models predicting the high vs. the low MDRS-22 score groups within the group of depressed patients. We also tested whether there were significant moderation effects of sex. We report B coefficients and validated the results using bias-corrected and accelerated bootstrap (1,000 resamples). P values < 0.05 were considered significant. Results related to the primary hypotheses are presented in Tables 3 and 4, which were included in the Bonferroni correction for multiple comparisons across 24 statistical models. P-values for secondary and exploratory hypotheses were not corrected for multiple testing; as no adjustment was applied, these results should be interpreted with caution. Additional Model 4: To complement the regression-based analyses, we conducted analyses of covariance (ANCOVAs) to examine group differences in mental health burden parameters at the level of adjusted group means. In these models, the SCL-90-R subscales served as the dependent variable, while group (HMD vs. LMD, HMD vs. HCS, LMD vs. HCS) was entered as fixed factors. Age, sex (and for depressed patients only BDI-II score) were included as covariates to control for the potential confounding effects. No interaction terms were included in the ANCOVA models. Estimated marginal means were calculated at the mean values of the covariates.

## Results

### Cohort characteristics

Table 1 shows a sociodemographic comparison between the HMD and LMD patient groups and the healthy control subjects. Relative to the depressed patients with LMD, the patients with HMD were younger (mean age: 36.4 vs. 45.7 years), and showed higher depression severity according to BDI-II (37.3 vs. 28.7). Table 2 shows the descriptive characteristics of MDRS-22 and SCL-90-R parameters for patient groups and healthy control subjects. 

For patients, the median MDRS-22 score was 1.60 (IQR 1.17–2.58) in men and 1.80 (IQR 1.17–2.28) in women, with no statistically significant difference between sexes (Mann-Whitney test, *p* = 0.912). Based on these values, the LMD group comprised 46 men with MDRS-22 scores ranging from 0.22 to 1.60 and 36 women with scores from 0.15 to 1.77, whereas the HMD group included 45 men with scores ranging from 1.70 to 5.44 and 36 women with scores from 1.82 to 5.78. Within the group of depressed patients with HMD, the MDRS-22 sum score did not correlate significantly with BDI-II or SCL-90-R scores except for the subscale anger-hostility (*N* = 79, *r* = 0.313, *P* = 0.005). The MDRS-22 sum score correlated with BDI-II scores in the group of patients with LMD (*N* = 81, *r* = 0.495, *P* < 0.001) and all SCL-90-R subscales (*N* = 80, r max 0.477, *P* ≤ 0.026). Correlations between the BDI-II and the SCL-90-R depression dimension were moderate to high across all subgroups (*r* = 0.31–0.91, all *p* < 0.001). Similarly, moderate to high correlations were observed for the SCL-90-R global indices (GSI: *r* = 0.45–0.92; PSDI: *r* = 0.36–0.90; PST: *r* = 0.39–0.88) and symptom dimensions, including somatization (*r* = 0.28–0.90), obsessive–compulsive symptoms (*r* = 0.35–0.89), interpersonal sensitivity (*r* = 0.45–0.93), anxiety (*r* = 0.39–0.85), anger–hostility (*r* = 0.21–0.87), phobic anxiety (*r* = 0.33–0.82), paranoid ideation (*r* = 0.34–0.89), and psychoticism (*r* = 0.39–0.84), with all correlations reaching statistical significance (*p* < 0.05). For the correlations’ descriptions and further correlations see Supplementary Table S2.

**Table 1:** Cohort characteristics.

	Patients with HMD (*N*_total_ = 81)			Patients with LMD (*N*_total_ = 82)			Healthy control subjects (*N*_total_ = 176)			Patients with HMD vs. patients with LMD		Patients with HMD vs. controls		Patients with LMD vs. controls	
	*N*	M/F	SD	*N*	M/F	SD	*N*	M/F	SD	χ² / t	*P*	χ² or t	*P*	χ² or t	*P*
% Men	81	56		82	56		176	49		< 0.1	0.944	1	0.319	1.2	0.279
Age (years)	81	36.4	14.1	82	45.7	14.6	176	37.2	13.7	-4.1	**< 0.001**	-0.4	0.684	4.5	**< 0.001**
% Partnered	79	48		77	55		176	68		0.6	0.421	9.3	**0.002**	4.3	**0.038**
% Married	79	28		80	48		175	27		6.5	**0.011**	< 0.1	0.945	9.9	**0.002**
% Divorced	79	14		78	21		176	14		1.2	0.274	< 0.1	0.953	1.6	0.207
BDI-II score	80	37.3	10.6	81	28.7	10.3	174	3.4	3.8	5.2	**< 0.001**	27.9	**< 0.001**	21.5	**< 0.001**
MDRS-22 score	81	2.6	0.8	82	1.1	0.4	176	0.4	0.4	15.2	**< 0.001**	24.2	**< 0.001**	14.7	**< 0.001**

The table shows the valid number of subjects analyzed (N), means (M), or relative frequencies (F), standard deviation (SD), and the results of χ²/Fisher and student’s t-tests. BDI-II Beck’s Depression Inventory-II, MDRS-22, Male Depression Risk Scale-22, HMD High Masculine Depression scores, LMD Low Masculine Depression scores. *P* < 0.05 in bold.

**Table 2:** Descriptive characteristics of MDRS-22 and SCL-90-R parameters within the patient groups and the healthy control subjects.

	Patients with HMD (*N*_total_ = 81)				Patients with LMD (*N*_total_ = 82)				Healthy Control Subjects (*N*_total_ = 176)			
	*N*	M	IQR (Q1-Q3)		*N*	M	IQR (Q1-Q3)		*N*	M	IQR (Q1-Q3)	
MDRS-22	81	2.4	2.1	3.0	82	1.2	0.8	1.5	176	0.3	0.1	0.6
GSI	79	80.0	76.6	80.0	80	68.1	61.1	79.8	176	43.6	41.6	46.7
PSDI	79	74.1	67.5	79.1	80	62.3	55.9	70.4	176	45.6	43.3	48.2
PST	79	74.8	67.4	77.6	80	64.7	58.6	71.6	176	43.2	39.8	47.6
Somatization	79	72.7	59.6	80.0	80	57.0	50.1	65.1	176	44.1	41.8	48.5
Obsessive-compulsive	79	80.0	74.9	80.0	80	77.0	66.9	80.0	176	44.1	41.4	49.2
Interpersonal sensibility	79	79.4	66.9	80.0	80	65.6	54.6	79.6	176	43.6	41.5	46.9
Depression	79	80.0	80.0	80.0	80	78.0	67.7	80.0	176	43.6	41.7	47.6
Anxiety	79	80.0	68.8	80.0	80	67.0	52.7	80.0	176	43.5	42.6	45.8
Anger-hostility	79	67.2	59.5	78.6	80	53.4	47.5	64.3	176	42.5	41.8	45.5
Phobic-anxiety	79	80.0	61.5	80.0	80	60.2	48.2	80.0	176	45.0	43.6	45.3
Paranoid ideation	79	65.8	56.7	79.6	80	52.3	45.2	65.4	176	43.0	41.1	47.4
Psychoticism	79	75.9	64.0	80.0	80	60.7	51.2	70.6	176	44.4	42.9	47.3
Processing time	72	436.0	321.0	536.5	76	495.5	335.5	618.8	158	276.5	222.3	332.3

The table shows the valid number of subjects analyzed (N), the medians (M), and the interquartile ranges (IQR). Values of the SCL-90-R test are shown as normalized T values with maximum values of 80. Median split of MDRS-22 scores used to separate patients into HMD and LMD subgroups: ≥1.70 for men, ≥ 1.82 for women. MDRS-22 Male Depression Risk Scale 22, SCL-90-R Symptom Checklist-90-Revised, HMD High Masculine Depression scores, LMD Low Masculine Depression scores, GSI Global Severity Index, PST Positive Symptom Total, PSDI Positive Symptom Distress Index.

### SCL-90-R scores

#### Patients with high masculine depression scores (HMD) vs. patients with low masculine depression scores (LMD)

**Fig. 1:**
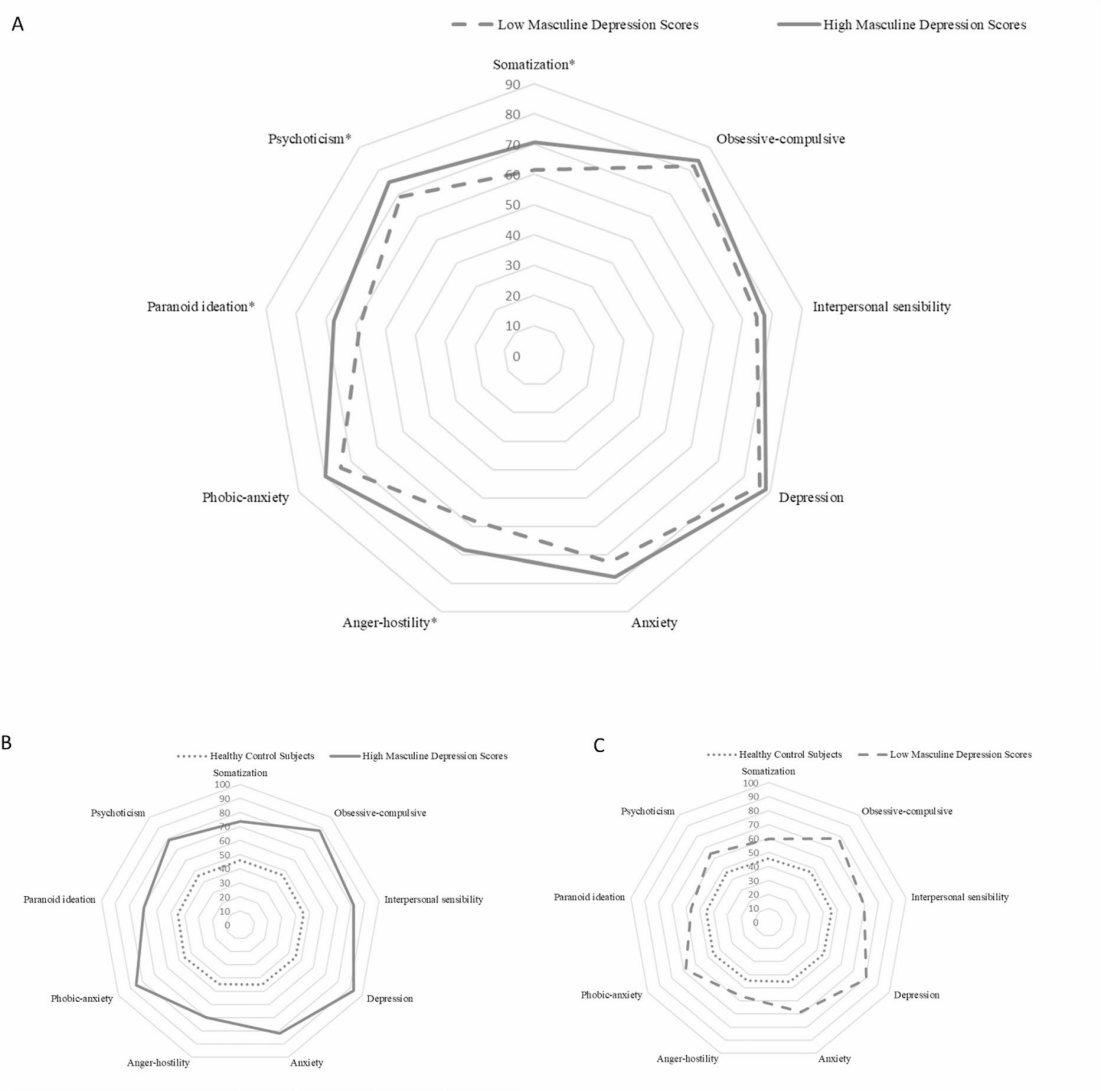
The figures show marginal means from ANCOVAs with the SCL-90-R values as the dependent variables. Median split of MDRS-22 scores used to separate patients into those with high vs. low masculine depression scores: ≥ 1.70 for men, ≥ 1.82 for women. (**A**) Group (patients with high vs. low masculine depression scores), Beck’s Depression Inventory-II score, sex, and age as predictors. *indicates significant differences. (**B**) Group (patients with high mascuine depression scores vs. healthy control subjects), sex, and age as predictors. (**C**) Group (patients with low masculine depression scores vs. healthy control subjects), sex, and age as predictors.

In regression models adjusted for sex, BDI-II scores, and age, the group of patients with HMD was associated with higher SCL-90-R GSI (B = 0.107, *P* < 0.001), PSDI (B = 0.071, *P* = 0.009) and PST (B = 0.087, *P* = 0.003) scores (Table 3). With focus on the SCL-90-R subcategories, the group of patients with HMD was associated with higher levels of somatization (B = 0.075, *P* < 0.001), anxiety (B = 0.036, *P* = 0.046), anger-hostility (B = 0.077, *P* < 0.001), phobic-anxiety (B = 0.033, *P* = 0.032), paranoid ideation (B = 0.060, *P* < 0.001), and psychoticism (B = 0.066, *P* < 0.001). Following Bonferroni correction for multiple comparisons across the primary hypotheses, only GSI, somatization, anger-hostility, paranoid ideation, and psychoticism remained statistically significant. In additional analyses of covariance adjusted for sex, BDI-II scores, and age, patients with HMD scores showed significantly higher adjusted mean levels of somatization, anger–hostility, paranoid ideation, and psychoticism compared with patients with LMD scores (Fig. 1A). There was no significant effect on the time required to complete the SCL-90-R, i.e., the total time participants took to respond to all 90 items. Mean response times were 277 s for HCS, 496 s for LMD, and 436 s for HMD. The group of patients with HMD was associated with higher BDI-II scores (B from 0.055 to 0.101, *P* from < 0.001 to 0.022; except for the model with SCL-90-R GSI value) and younger age (B from − 0.059 to − 0.040, *P * < 0.001). In the statistical moderation analyses, no significant effect was found for the interaction between the SCL-90-R variables and sex (Table 3). Despite the absence of significant sex-by-SCL-90-R interactions in the moderation analyses, additional regression models were run separately for men and women and are reported as exploratory descriptive analyses. In our sex-seperated analyzes, the group of patients with HMD was associated with higher values for somatization, anger-hostility, and paranoid ideation (Supplementary Tables S3 and Supplementary Fig. 1) in male patients. In the female patients, this effect was present for higher values of SCL-90-R GSI, PST, somatization, anger-hostility, paranoid ideation, and psychoticism (Supplementary Tables S4 and Supplementary Fig. 1).

#### MDRS-22 scores

Within the group of depressed patients, higher SCL-90-R GSI (B = 0.028, *P* = 0.004), PSDI (B = 0.020, *P* = 0.032), and PST (B = 0.032, *P* < 0.001) scores were associated with higher MDRS-22 scores. Within the SCL-90-R subcategories somatization (B = 0.027, *P* < 0.001), anxiety (B = 0.013, *P* = 0.038), anger-hostility (B = 0.033, *P* < 0.001), phobic-anxiety (B = 0.009, *P* = 0.092), paranoid ideation (B = 0.022, *P* < 0.001), and psychoticism (B = 0.023, *P* < 0.001) were related to higher MDRS-22 scores. After applying Bonferroni correction for multiple testing across the primary hypotheses, only PST, somatization, anger-hostility, paranoid ideation, and psychoticism remained statistically significant. Higher BDI-II scores (B = from 0.024 to 0.043, *P*  from < 0.001 to 0.004) and younger age (B = from − 0.018 to − 0.013, *P* from < 0.001 to 0.003) were associated with higher MDRS-22 scores (Table 4).

#### Patients with HMD vs. healthy control subjects and patients with LMD vs. healthy control subjects

The group of patients with HMD as well as the group of patients with LMD (vs. the group of healthy controls) were significantly associated with all SCL-90-R subscales and the completion time in the models taking into account sex and age as covariates (Supplementary Tables S5 and S6).

**Table 3:** Binary logistic regression to differentiate between patients with High Masculine Depression scores and patients with Low Masculine Depression scores using the SCL 90-R values as primary predictors in separate models adjusted for sex, BDI-II and age in depressed patients only.

Dependent Variable: HMD vs. LMD patients		Primary predictor			Sex			BDI-II			Age			Primary predictor-by sex †
	*N*	B	Wald	*P*	B	Wald	*P*	B	Wald	*P*	B	Wald	*P*	*P*
Primary predictor: SCL-90-Values														
GSI	158	0.107	11.071	**0.001**^#,^*	-0.285	0.465	0.495	0.041	2.823	0.093	-0.048	12.921	< 0.001*	0.723
PSDI	158	0.071	6.801	0.009*	-0.559	1.963	0.161	0.055	5.207	0.022*	-0.043	10.474	< 0.001*	0.847
PST	158	0.087	8.588	0.003*	-0.263	0.382	0.536	0.058	6.289	0.012*	-0.048	12.984	< 0.001*	0.527
Somatization	158	0.075	15.608	**< 0.001**^#,^*	-0.777	3.444	0.063	0.068	9.915	0.002*	-0.040	8.886	< 0.001*	0.451
Obsessive-compulsive	158	0.028	0.921	0.337	-0.581	2.124	0.145	0.084	14.844	< 0.001*	-0.049	14.119	< 0.001*	0.290
Interpersonal sensibility	158	0.024	1.378	0.240	-0.514	1.594	0.207	0.076	9.752	0.002*	-0.048	13.924	< 0.001*	0.897
Depression	158	0.063	2.756	0.097	-0.388	0.857	0.354	0.074	10.726	0.001*	-0.046	12.171	< 0.001*	0.738
Anxiety	158	0.036	3.993	0.046*	-0.548	1.869	0.172	0.073	10.749	0.001*	-0.049	14.339	< 0.001*	0.997
Anger-hostility	158	0.077	16.870	**< 0.001**^#,^*	-0.516	1.488	0.223	0.068	10.214	0.001*	-0.057	15.395	< 0.001*	0.417
Phobic-anxiety	158	0.033	4.615	0.032*	-0.588	2.177	0.140	0.076	12.565	< 0.001*	-0.047	12.903	< 0.001*	0.815
Paranoid ideation	158	0.060	12.129	**< 0.001**^#,^*	-0.337	0.671	0.413	0.061	7.954	0.005*	-0.055	15.490	< 0.001*	0.726
Psychoticism	158	0.066	11.034	**0.001**^#,^*	-0.433	1.111	0.292	0.060	7.051	0.008*	-0.048	13.035	< 0.001*	0.709
Processing time	147	-0.001	1.289	0.256	-0.813	3.604	0.058	0.101	19.829	< 0.001*	-0.059	15.344	< 0.001*	0.710

The table shows the valid number of subjects analyzed (N) and the results of binary logistic regression analyses. Bold p-values indicate nominal significance (*p* < 0.05). ^#^ indicates significance after Bonferroni correction across the 12 predictors. *indicates *p* < 0.05 in bootstrap analysis. Covariates were not included in the multiple testing correction. †Independent models computed used to determine the interaction parameters. Median split of MDRS-22 scores used to separate patients into HMD and LMD subgroups: ≥1.70 for men, ≥ 1.82 for women. SCL-90-R Symptom Checklist-90-Revised, BDI-II Beck Depression Inventory-II, HMD High Masculine Depression scores, LMD Low Masculine Depression scores, GSI Global Severity Index, PST Positive Symptom Total, PSDI Positive Symptom Distress Index. Coding: Patients with Low Masculine Depression scores = 0 vs. Patients with High Masculine Depression scores = 1; Males = 1 vs. Females = 2.

**Table 4:** Linear regression to predict Male Depression Risk Scale-22 scores using the SCL-90-R values as primary predictors in separate models adjusted for sex, BDI-II and age in depressed patients only.

		Primary predictor			Sex			BDI-II			Age		
	*N*	B	T	*P*	B	T	*P*	B	T	*P*	B	T	*P*
Dependent Variable: Male Depression Risk Scale-22 scores													
Primary predictor: SCL-90-R Values													
GSI	158	0.028	2.938	0.004*	– 0.184	– 1.305	0.194	0.024	2.916	0.004*	– 0.015	– 3.536	0.001*
PSDI	158	0.020	2.160	0.032*	– 0.280	– 2.042	0.043	0.029	3.537	0.001*	– 0.014	– 3.181	0.002*
PST	158	0.032	3.388	**0.001**^#,^*	– 0.141	– 0.990	0.324	0.025	3.302	0.001*	– 0.015	– 3.557	0.001*
Somatization	158	0.027	4.588	**< 0.001**^#,^*	– 0.335	– 2.575	0.011	0.028	4.437	< 0.001*	– 0.013	– 3.020	0.003*
Obsessive-compulsive	158	0.003	0.296	0.768	– 0.300	– 2.116	0.036	0.040	5.507	< 0.001*	– 0.017	– 3.734	< 0.001*
Interpersonal sensibility	158	0.010	1.382	0.169	– 0.251	– 1.743	0.083	0.033	4.155	< 0.001*	– 0.017	– 3.743	< 0.001*
Depression	158	0.015	1.311	0.192	– 0.238	– 1.610	0.109	0.035	4.512	< 0.001*	– 0.015	– 3.393	0.001*
Anxiety	158	0.013	2.094	0.038*	– 0.262	– 1.889	0.061	0.032	4.497	< 0.001*	– 0.017	– 3.822	< 0.001*
Anger-hostility	158	0.033	6.083	**< 0.001**^#,^*	– 0.190	– 1.509	0.133	0.025	4.167	< 0.001*	– 0.017	– 4.228	< 0.001*
Phobic-anxiety	158	0.009	1.696	0.092	– 0.286	– 2.078	0.039	0.035	5.071	< 0.001*	– 0.016	– 3.581	< 0.001*
Paranoid ideation	158	0.022	3.922	**< 0.001**^#,^*	– 0.201	– 1.489	0.139	0.027	3.888	< 0.001*	– 0.018	– 4.131	< 0.001*
Psychoticism	158	0.023	3.593	**< 0.001**^#,^*	– 0.218	– 1.606	0.110	0.026	3.619	< 0.001*	– 0.015	– 3.574	< 0.001*
Processing time	147	0.000	– 0.320	0.750	– 0.345	– 2.357	0.020	0.043	6.498	< 0.001*	– 0.017	– 3.347	0.001*

The table shows the valid number of subjects analyzed (N) and the results of linear regression analyses. Bold p-values indicate nominal significance (*p* < 0.05). ^#^ indicates significance after Bonferroni correction across the 12 predictors. *indicates *p* < 0.05 in bootstrap analysis. Covariates were not included in the multiple testing correction. SCL-90-R Symptom Checklist-90-Revised, BDI-II Beck Depression Inventory-II, HMD High Masculine Depression scores, LMD Low Masculine Depression scores, GSI Global Severity Index, PST Positive Symptom Total, PSDI Positive Symptom Distress Index. Coding: Males = 1, Females = 2.

## Discussion

In this work, we analyzed associations of mental health burden with high versus low masculine depression scores and no depression. In our patient cohort, 72 female and 91 male patients were subdivided into two groups applying a sex-specific median split. Here we found that patients with HMD scores (in comparison to patients with LMD scores) as well as higher MDRS-22 scores were associated with higher acute mental health burden measured by SCL-90-R, even after adjusting for overall depression severity. This was appropriate for the three global dimensions GSI, PST, and PSDI. However, following Bonferroni correction for multiple comparisons, only the dimension GSI (PST in the linear model) remained statistically significant. Completion time did not significantly differ between HMD and LMD subgroups within the depressed sample, despite differences in reported symptom burden. The ability to perform the task at a comparable pace may reflect functional coping strategies or a tendency to suppress internal distress in patients with HMD. Together with prior findings from the same cohort indicating a lower utilization of mental health care services among these patients^13^, these observations do not allow for causal inferences. Rather, the findings may reflect a bidirectional relationship between higher masculine depression symptoms and broader psychopathology, in which sustained functioning, coping strategies, or tendencies to minimize or externalize internal distress may coexist with elevated psychological burden. However, these interpretations remain speculative, as no direct measures of coping, emotional suppression, or work-related functioning were included in the present study.

With focus on the subcategories, we found that the dimensions somatization, anxiety, anger-hostility, paranoid ideation, and psychoticism were associated with the group of patients with HMD (vs. LMD) as well as higher MDRS-22 scores in our validation analyses using different statical models. After Bonferroni correction for multiple comparisons, only somatization, anger-hostility, paranoid ideation, and psychoticism remained significant in both sets of analyses. These dimensions were associated with the masculine depression spectrum even after statistical adjustment for overall depression severity, while not implying full independence from depressive symptom severity. That is partly consistent with our former study in the same cohort showing patients with HMD exhibited more pronounced personality accentuations including impulsive, borderline, and dissocial dimensions than patients with LMD scores^11^. In this former analyses, patients with HMD also scored higher on the paranoid, schizotypal, and anxious dimensions with these effects not persisting after adjustment for multiple hypothesis testing^11^.

Previous studies have suggested links between anxiety, depressive symptoms, and substance use^27–29^. Prior work in our cohort indicated that HMD is associated with more frequent alcohol use (including binge drinking), tobacco, and illicit drug use, which may be preferred over seeking professional help or contacting a physician when experiencing elevated stress; however, the study design does not allow for causal conclusions^13^. These findings provide background context for our results, but the same limitation applies to the present analysis. While Haynes et al.^30^ showed in a cross-sectional survey that excessive alcohol consumption was not associated with the onset of anxiety and depression and sub-threshold symptoms were only weakly associated with new-onset alcohol dependence, personality traits such as borderline personality disorder have been associated with both substance misuse and anxiety^31,32^. Additionally, substance use disorders or antisocial behavior have been considered potential factors promoting externalizing behaviors^33,34^. Externalizing behaviors, which are characteristic of the so-called masculine depression, may be promoted by chronic stressors. Evidence for these patterns has also been observed in prior populations, such as female university students^10^. Furthermore, previous work in our own cohort indicated that HMD was associated with longer working hours compared with healthy controls^13^. In addition, overwork has been linked to negative health outcomes^16^. Nonetheless, the connection between stress experience, longer working hours, and the occurrence of psychiatric illnesses is still unclear^35^. In our current analyses, however, no significant associations between anxiety or phobic anxiety and higher MDRS-22 scores were observed after correction for multiple testing.

While the present study demonstrates that patients with HMD scores exhibit higher mental health burden than those with LMD scores, it is important to recognize that both the MDRS-22 and SCL-90-R rely on self-reported psychopathology, which may contribute to overlapping variance. Future studies integrating biological markers (e.g., stress hormones, inflammatory parameters) or behavioral measures (e.g., cognitive performance tests, real-world help-seeking behavior) could help delineate the HMD construct more mechanistically and clarify pathways linking these symptoms to broader psychopathology.

Interestingly, we did not find a significant effect of the interaction between the SCL-90R variables and sex. This is consistent with our observation that male depressed patients did not score higher on the MDRS-22 scale than female patients. Similarly, Möller-Leimkühler and Yücel^10^ reported that masculine depression is also highly prevalent among female university students. These findings suggest that the construct of masculine depression may capture symptom patterns that are not strictly sex-specific. While the term “masculine depression” emphasizes traits historically associated with men, our results indicate that these symptom constellations can also occur in women, highlighting the need to interpret the construct as reflecting a particular depressive profile. Accordingly, the term “masculine depression” should be understood as a descriptive label for a specific symptom profile characterized by emotional suppression, somatic symptoms, drug and alcohol use, anger, aggression, and risk-taking, rather than implying inherent sex specificity. Recent work argues for replacing the oversimplified binary approach to sex by deconstructing it and using dimensional parameters^36,37^. This approach warrants consensus on an alternative name for “masculine depression”. It might therefore be clearer and more accurate to frame the study as an investigation of externalized symptom expression rather than a distinct masculine subtype.

## Strengths and limitations

Our results do not allow for conclusions regarding causality or directionality, due to the associational study design. The symptoms described in patients with HMD scores may represent relatively independent features of masculine depression or, alternatively, act as risk factors or correlates rather than consequences. Future longitudinal studies are therefore required to disentangle causal pathways and temporal relationships. Furthermore, our sample consisted exclusively of individuals undergoing inpatient psychiatric treatment. Given evidence of reduced help-seeking behavior among individuals with elevated masculine depression symptoms, severely affected non–treatment-seeking individuals were likely underrepresented. As a result, our findings may underestimate the true psychological burden and symptom severity associated with masculine depression. Accordingly, the results primarily characterize its manifestation in a treatment-seeking inpatient population, limiting generalizability to the broader community. Future longitudinal studies should take this into greater account when selecting participants. Although overlaps with anxiety disorders and personality accentuations were observed, presumed differences in pathogenesis—particularly regarding value orientation and work-related stress—support the continued use of the concept of masculine depression as a heuristic framework to facilitate more targeted clinical understanding and intervention. The restriction to a BMI range of 18.5–35 kg/m² may limit generalizability to individuals with extreme underweight or obesity. As no correction for multiple testing was applied for secondary and exploratory hypotheses, the reported nominal p-values should be interpreted with caution as they could reflect chance. Finally, we acknowledge that the term masculine depression attributes certain externalizing symptoms (e.g., aggression) to masculinity, which we consider conceptually problematic given that such behaviors are often perceived as socially maladaptive. In the absence of a more precise, gender-independent terminology that better reflects role-related or contextual demands, the term is used here pragmatically and should not be understood as inherently linked to biological sex.

Our project includes a sex-balanced and large study cohort which is a major strength. We analyzed relevant influencing factors such as sex, BDI-II, and age in the statistical models.

## Conclusion

This study indicates that so-called masculine depression is associated with a substantial mental health burden beyond overall depression severity within a treatment-seeking inpatient population. Importantly, due to the selective nature of the sample, these findings likely represent a conservative estimate of the psychological burden associated with masculine depression, as severely affected individuals who do not seek help may be underrepresented. While the results therefore cannot be generalized to the full community manifestation of masculine depression, they nonetheless highlight its clinical relevance and underscore the need for early, specialized, and low-threshold support services aimed at reaching individuals who may otherwise remain untreated.

## Supplementary Information

Below is the link to the electronic supplementary material.

Supplementary Material 1

## Data Availability

Data are available upon request.

## Abbreviations

BDI-II: Beck Depression Inventory-II
BMI: Body Mass Index
DSM: Diagnostic and Statistical Manual of Mental Disorders
GMDS: Gotland Male Depression Scale
GSI: Global Severity Index
HAMD-17: Hamilton Rating Scale for Depression-17
HMD: High masculine depression scores
ICD: International Classification of Disease
IQR: Interquartile ranges
LMD: Low masculine depression scores
MDRS-22: Male Depression Risk Scale-22
PSDI: Positive Symptom Distress Index
PST: Positive Symptom Total
SCL-90-R: Symptom Checklist-90-Revised
