# Supplementary material for: Masculine depression and acute mental health burden
Source: Sci Rep. 2026 Apr 5;16:11606. doi: 10.1038/s41598-026-44727-7 (PMC13057464; doi:10.1038/s41598-026-44727-7)
Supplement: Supplementary file 1 — Supplementary Material 1 [file 41598_2026_44727_MOESM1_ESM.docx]

**Supplementary Material**

**Masculine Depression and Acute Mental Health Burden**

Claudia von Zimmermann*^,1^, Christian Weinland^1^, Johannes Kornhuber^1^, Bernd Lenz^1,2^, Christiane Mühle^1^

^1^Department of Psychiatry and Psychotherapy, Friedrich-Alexander University Erlangen-Nürnberg (FAU), Germany.

^2^Department of Addictive Behavior and Addiction Medicine, Central Institute of Mental Health (CIMH), Medical Faculty Mannheim, Heidelberg University, Germany.

*Corresponding author. E-Mail: Claudia.von.Zimmermann@uk-erlangen.de

**Supplementary Figure 1**

**Supplementary Table S1** Items of the MDRS-22 translated into German

**Supplementary Table S2** Pearson correlations among variables

**Supplementary Table S3** Binary logistic regression to differentiate between patients with high masculine depression scores and patients with low masculine depression scores in male patients using the SCL-90-R values as primary predictors adjusted for BDI-II and age

**Supplementary Table S4** Binary logistic regression to differentiate between patients with high masculine depression scores and patients with low masculine depression scores in female patients using the SCL-90-R values as primary predictors adjusted for BDI-II and age

**Supplementary Table S5** Binary logistic regression to differentiate between patients with high masculine depression scores and healthy control subjects using the SCL-90-R as primary predictor adjusted for sex and age

**Supplementary Table S6** Binary logistic regression to differentiate between patients with low masculine depression scores and healthy control subjects using the SCL-90-R as primary predictor adjusted for sex and age


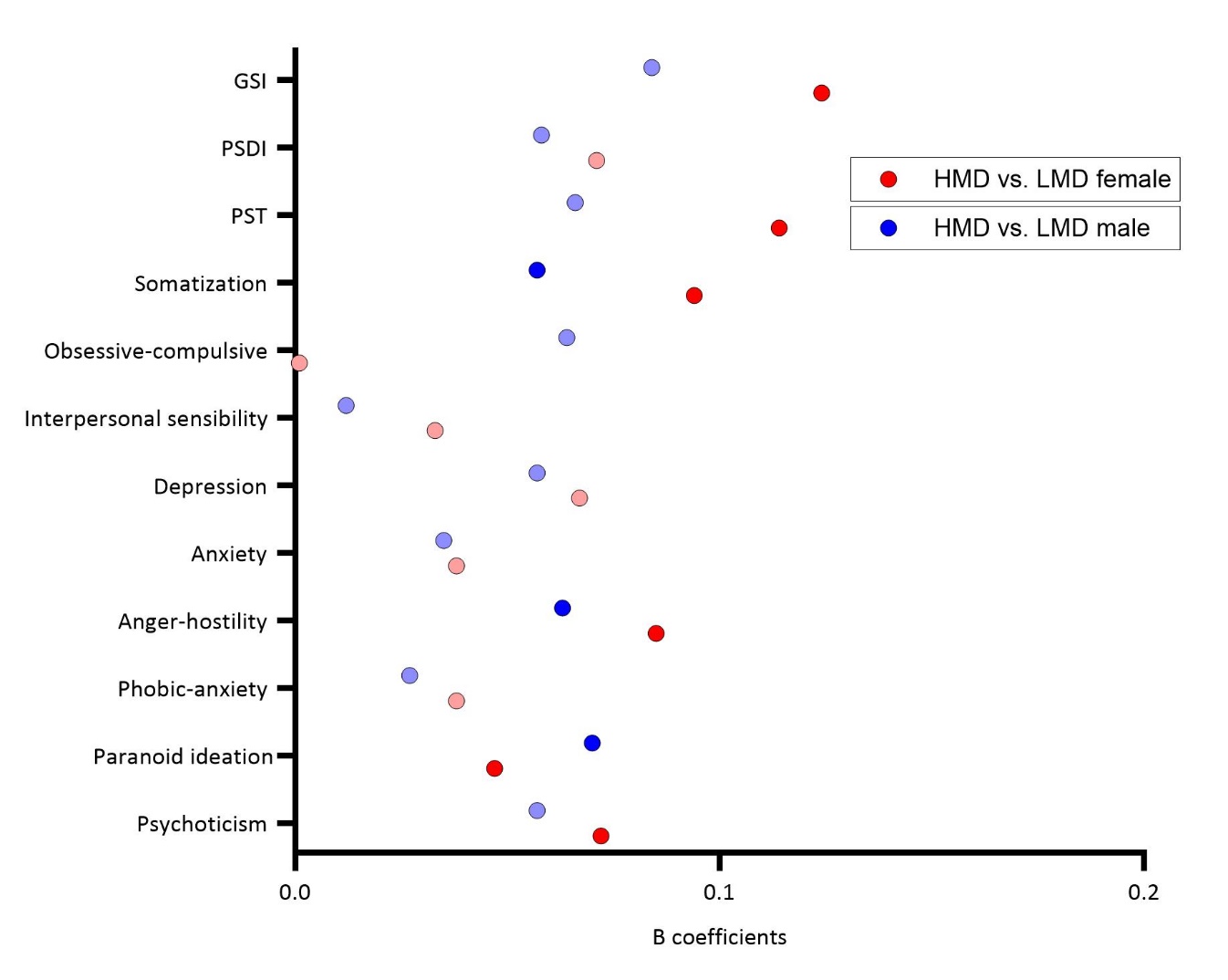
 Caption Supplementary Figure 1. The figure shows B coefficients from binary logistic regression analyses to predict group (i.e. patients with HMD vs. patients with LMD scores). HMD High Masculine Depression scores, LMD Low Masculine Depression scores. B coefficients reaching statistical significance are indicated by non-transparent markers.

| **Supplementary Table S1** Items of the MDRS-22 translated into German |
| --- |
| 1.Ich habe meine negativen Gefühle unterdrückt.  2.Ich habe versucht meine Niedergeschlagenheit zu verdrängen.  3.Ich habe meine Probleme verborgen.  4. Ich habe alles alleine lösen müssen.  5. Ich habe Drogen genommen, um zurecht zu kommen.  6. Drogengebrauch hat mir vorrübergehende Erleichterung verschafft.  7. Ich habe Drogen beschafft.  8. Ich habe Alkohol gebraucht, um herunterzukommen.  9. Ich habe leichten Zugang zu Alkohol gebraucht.  10. Ich trank mehr Alkohol als sonst.  11. Ich habe mich durchs Trinken nicht mehr so schlecht gefühlt.  12. Ich verhielt mich anderen gegenüber verbal aggressiv.  13. Ich bin andere verbal angegangen obwohl ich nicht provoziert worden bin.  14. Es war schwierig meine Wut/Ärger unter Kontrolle zu haben.  15. Ich habe in Situationen aggressiv überreagiert.  16. Ich hatte unerklärliche Schmerzen.  17. Ich hatte Magenschmerzen.  18. Ich hatte regelmäßige Kopfschmerzen.  19. Ich litt häufiger unter Sodbrennen als sonst.  20. Ich bin gefährlicher oder aggressiver Auto gefahren.  21. Ich habe aufgehört über die Folgen meiner Handlungen nachzudenken.  22. Ich habe unnötige Risiken in Kauf genommen. |
| The table shows the translated German version of the Male Depression Rating Scale-22 (MDRS-22) [1]. The questionnaire items listed in Table 1 have been previously published in von Zimmermann et al, 2023[2]. |

| **Supplementary Table S2** Pearson correlations among variables | | | | | | | | | | | | | | | | |
| --- | --- | --- | --- | --- | --- | --- | --- | --- | --- | --- | --- | --- | --- | --- | --- | --- |
| Correlation with | BDI-II / MDRS | Age | Sex | GSI | PSDI | PST | Somati-zation | Obsess-ive-compul-sive | Interper-sonal sensibi-lity | Depress-ion | Anxiety | Anger-hostility | Phobic-anxiety | Paranoid ideation | Psychoti-cism | Process-ing time |
| **BDI-II** |  |  |  |  |  |  |  |  |  |  |  |  |  |  |  |  |
| **HMD** |  |  |  |  |  |  |  |  |  |  |  |  |  |  |  |  |
| r | 0.161 | 0.168 | .312** | .449** | .499** | .394** | .276* | .350** | .517** | .308** | .385** | 0.211 | .330** | .335** | .391** | 0.011 |
| P | 0.153 | 0.136 | **0.005** | **< 0.001** | **< 0.001** | **< 0.001** | **0.015** | **0.002** | **< 0.001** | **0.006** | **< 0.001** | **0.064** | **0.003** | **0.003** | **< 0.001** | 0.926 |
| N | 80 | 80 | 80 | 78 | 78 | 78 | 78 | 78 | 78 | 78 | 78 | 78 | 78 | 78 | 78 | 71 |
| **LMD** |  |  |  |  |  |  |  |  |  |  |  |  |  |  |  |  |
| r | .495** | -0.055 | .241* | .682** | .686** | .536** | .375** | .502** | .595** | .578** | .509** | .318** | .439** | .435** | .511** | 0.155 |
| P | **< .001** | 0.624 | **0.030** | **< 0.001** | **< 0.001** | **< 0.001** | **0.001** | **< 0.001** | **< 0.001** | **< 0.001** | **< 0.001** | **0.004** | **< 0.001** | **< 0.001** | < **0.001** | 0.181 |
| N | 81 | 81 | 81 | 80 | 80 | 80 | 80 | 80 | 80 | 80 | 80 | 80 | 80 | 80 | 80 | 76 |
| **HMD + LMD** |  |  |  |  |  |  |  |  |  |  |  |  |  |  |  |  |
| r | .445** | -0.069 | .257** | .652** | .669** | .558** | .450** | .484** | .610** | .537** | .523** | .391** | .472** | .485** | .550** | 0.035 |
| P | **< 0.001** | 0.387 | **0.001** | **< 0.001** | **< 0.001** | **< 0.001** | **< 0.001** | **< 0.001** | **< 0.001** | **< 0.001** | **< 0.001** | **< 0.001** | **< 0.001** | **< 0.001** | **< 0.001** | 0.670 |
| N | 161 | 161 | 161 | 158 | 158 | 158 | 158 | 158 | 158 | 158 | 158 | 158 | 158 | 158 | 158 | 147 |
| **HCS** |  |  |  |  |  |  |  |  |  |  |  |  |  |  |  |  |
| r | .603** | -0.062 | -0.103 | .665** | .357** | .644** | .393** | .597** | .449** | .677** | .477** | .399** | .413** | .521** | .464** | .223** |
| P | **< 0.001** | 0.413 | 0.175 | **< 0.001** | **< 0.001** | **< 0.001** | **< 0.001** | **< 0.001** | **< 0.001** | **< 0.001** | **< 0.001** | **< 0.001** | **< 0.001** | **< 0.001** | **< 0.001** | **0.005** |
| N | 174 | 174 | 174 | 174 | 174 | 174 | 174 | 174 | 174 | 174 | 174 | 174 | 174 | 174 | 174 | 157 |
| **All participants** |  |  |  |  |  |  |  |  |  |  |  |  |  |  |  |  |
| r | .783** | 0.094 | 0.002 | .917** | .865** | .879** | .764** | .878** | .855** | .906** | .845** | .739** | .796** | .736** | .836** | .482** |
| P | **< 0.001** | 0.085 | 0.968 | **< 0.001** | **< 0.001** | **< 0.001** | **< 0.001** | **< 0.001** | **< 0.001** | **< 0.001** | **< 0.001** | **< 0.001** | **< 0.001** | **< 0.001** | **< 0.001** | **< 0.001** |
| N | 335 | 335 | 335 | 332 | 332 | 332 | 332 | 332 | 332 | 332 | 332 | 332 | 332 | 332 | 332 | 304 |
| **HMD +  HCS** |  |  |  |  |  |  |  |  |  |  |  |  |  |  |  |  |
| r | 0.007 | -0.019 | .932** | .876** | .899** | .830** | .897** | .888** | .926** | .888** | .819** | .871** | .815** | .891** | .550** | 0.035 |
| P | 0.913 | 0.758 | **< 0.001** | **< 0.001** | **< 0.001** | **< 0.001** | **< 0.001** | **< 0.001** | **< 0.001** | **< 0.001** | **< 0.001** | **< 0.001** | **< 0.001** | **< 0.001** | **< 0.001** | 0.670 |
| N | 254 | 254 | 252 | 252 | 252 | 252 | 252 | 252 | 252 | 252 | 252 | 252 | 252 | 252 | 158 | 147 |
| **LMD +  HCS** |  |  |  |  |  |  |  |  |  |  |  |  |  |  |  |  |
| r | .221** | -0.027 | .908** | .813** | .850** | .681** | .868** | .806** | .906** | .809** | .660** | .740** | .643** | .780** | .550** | 0.035 |
| P | **< 0.001** | 0.668 | **< 0.001** | **< 0.001** | **< 0.001** | **< 0.001** | **< 0.001** | **< 0.001** | **< 0.001** | **< 0.001** | **< 0.001** | **< 0.001** | **< 0.001** | **< 0.001** | **< 0.001** | 0.670 |
| N | 255 | 255 | 254 | 254 | 254 | 254 | 254 | 254 | 254 | 254 | 254 | 254 | 254 | 254 | 158 | 147 |
| **MDRS** |  |  |  |  |  |  |  |  |  |  |  |  |  |  |  |  |
| **HMD** |  |  |  |  |  |  |  |  |  |  |  |  |  |  |  |  |
| r | 0.161 | -0.149 | -0.085 | 0.090 | 0.112 | 0.185 | 0.218 | -0.009 | 0.079 | 0.047 | 0.122 | .313** | 0.086 | 0.140 | 0.169 | 0.051 |
| P | 0.153 | 0.184 | 0.452 | 0.429 | 0.325 | 0.102 | **0.054** | 0.939 | 0.488 | 0.681 | 0.284 | **0.005** | 0.452 | 0.217 | 0.138 | 0.668 |
| N | 80 | 81 | 81 | 79 | 79 | 79 | 79 | 79 | 79 | 79 | 79 | 79 | 79 | 79 | 79 | 72 |
| **LMD** |  |  |  |  |  |  |  |  |  |  |  |  |  |  |  |  |
| r | .495** | 0.049 | 0.062 | .466** | .361** | .477** | .261* | .315** | .460** | .463** | .330** | .409** | .248* | .426** | .419** | 0.102 |
| P | **< 0.001** | 0.661 | 0.581 | **< 0.001** | **0.001** | **< 0.001** | **0.020** | **0.004** | **< 0.001** | **< 0.001** | **0.003** | **< 0.001** | **0.026** | **< 0.001** | **< 0.001** | 0.380 |
| N | 81 | 82 | 82 | 80 | 80 | 80 | 80 | 80 | 80 | 80 | 80 | 80 | 80 | 80 | 80 | 76 |
| **HCS** |  |  |  |  |  |  |  |  |  |  |  |  |  |  |  |  |
| r | .603** | 0.060 | -0.088 | .734** | .299** | .732** | .485** | .621** | .567** | .635** | .538** | .469** | .491** | .653** | .622** | .275** |
| P | **< 0.001** | 0.432 | 0.245 | **< 0.001** | **< 0.001** | **< 0.001** | **< 0.001** | **< 0.001** | **< 0.001** | **< 0.001** | **< 0.001** | **< 0.001** | **< 0.001** | **< 0.001** | **< 0.001** | **< 0.001** |
| N | 174 | 176 | 176 | 176 | 176 | 176 | 176 | 176 | 176 | 176 | 176 | 176 | 176 | 176 | 176 | 158 |
| The table shows the valid number of subjects analyzed (N) and the results of Pearson correlation analyses (r). Median split of MDRS-22 scores used to separate patients into HMD and LMD subgroups: ≥1.70 for men, ≥1.82 for women. BDI-II Beck’s Depression Inventory-II; MDRS-22 Male Depression Rating Scale-22, HMD High Masculine Depression scores, LMD Low Masculine Depression scores, HCS Healthy control subjects, GSI Global Severity Index, PST Positive Symptom Total, PSDI Positive Symptom Distress Index. P < 0.05 in bold. **The correlation is significant at the 0.01 level (two-tailed). | | | | | | | | | | | | | | | | |

| **Supplementary Table S3** Binary logistic regression to differentiate between patients with high masculine depression scores and patients with low masculine depression scores in **male** patients using the SCL-90-R values as primary predictors adjusted for BDI-II and age | | | | | | | | | | |  |
| --- | --- | --- | --- | --- | --- | --- | --- | --- | --- | --- | --- |
|  |  | Primary predictor | | | BDI-II | | | Age | | | |
|  | N | B | Wald | P | B | Wald | P | B | Wald | P | |
| **Dependent Variable: Patients with high masculine depression scores vs. Patients with low masculine depression scores** |  |  |  |  |  |  |  |  |  |  | |
| **Primary predictor: SCL-90-R values** |  |  |  |  |  |  |  |  |  |  | |
| GSI | 88 | 0.084 | 3.434 | 0.064* | 0.074 | 3.624 | 0.057* | -0.069 | 12.510 | **< 0.001*** | |
| PSDI | 88 | 0.058 | 2.014 | 0.156 | 0.085 | 4.662 | **0.031*** | -0.063 | 9.755 | **0.002*** | |
| PST | 88 | 0.066 | 2.836 | 0.092 | 0.086 | 5.286 | **0.021*** | -0.070 | 12.851 | **< 0.001*** | |
| Somatization | 88 | 0.057 | 4.954 | **0.026*** | 0.093 | 7.362 | **0.007*** | -0.063 | 10.101 | **0.001*** | |
| Obsessive-compulsive | 88 | 0.064 | 1.863 | 0.172 | 0.099 | 8.296 | **0.004*** | -0.074 | 14.199 | **< 0.001*** | |
| Interpersonal sensibility | 88 | 0.012 | 0.153 | 0.696 | 0.110 | 7.395 | **0.007*** | -0.072 | 13.570 | **< 0.001*** | |
| Depression | 88 | 0.057 | 0.713 | 0.398 | 0.107 | 8.982 | **0.003*** | -0.069 | 12.168 | **< 0.001*** | |
| Anxiety | 88 | 0.035 | 2.014 | 0.156 | 0.096 | 7.295 | **0.007*** | -0.074 | 14.129 | **< 0.001*** | |
| Anger-hostility | 88 | 0.063 | 5.878 | **0.015*** | 0.084 | 5.759 | **0.016*** | -0.071 | 11.912 | **0.001*** | |
| Phobic-anxiety | 88 | 0.027 | 1.548 | 0.213 | 0.100 | 7.788 | **0.005*** | -0.071 | 12.936 | **< 0.001*** | |
| Paranoid ideation | 88 | 0.070 | 5.941 | **0.015*** | 0.064 | 2.713 | 0.100 | -0.073 | 13.048 | **< 0.001*** | |
| Psychoticism | 88 | 0.057 | 3.689 | 0.055* | 0.081 | 4.694 | **0.030*** | -0.071 | 13.356 | **< 0.001*** | |
| Processing time | 83 | -0.001 | 0.212 | 0.645 | 0.124 | 11.201 | **0.001*** | -0.075 | 12.987 | **< 0.001*** | |
| The table shows the valid number of subjects analyzed (N) and the results of binary logistic regression analyses. Median split of MDRS-22 scores used to separate patients into HMD and LMD subgroups: ≥1.70 for men, ≥1.82 for women. SCL-90-R Symptom Checklist-90-Revised, BDI-II Beck Depression Inventory-II, GSI Global Severity Index, PST Positive Symptom Total, PSDI Positive Symptom Distress Index. P < 0.05 in bold, * significant in bootstrap analysis. Coding: Patients with low masculine depression scores = 0 vs. Patients with high masculine depression scores = 1. | | | | | | | | | | |  |

| **Supplementary Table S4** Binary logistic regression to differentiate between patients with high masculine depression scores and patients with low masculine depression scores in **female** patients using the SCL-90-R values as primary predictors adjusted for BDI-II and age | | | | | | | | | | |  |
| --- | --- | --- | --- | --- | --- | --- | --- | --- | --- | --- | --- |
|  |  | Primary predictor | | | BDI-II | | | Age | | | |
|  | N | B | Wald | P | B | Wald | P | B | Wald | P | |
| **Dependent Variable: Patients with high masculine depression scores vs. Patients with low masculine depression scores** |  |  |  |  |  |  |  |  |  |  | |
| **Primary predictor: SCL-90-R values** |  |  |  |  |  |  |  |  |  |  | |
| GSI | 70 | 0.124 | 6.919 | **0.009*** | 0.022 | 0.417 | 0.518 | -0.026 | 1.702 | 0.192 | |
| PSDI | 70 | 0.071 | 3.364 | 0.067 | 0.043 | 1.784 | 0.182 | -0.025 | 1.662 | 0.197 | |
| PST | 70 | 0.114 | 5.724 | **0.017*** | 0.041 | 1.813 | 0.178 | -0.023 | 1.418 | 0.234 | |
| Somatization | 70 | 0.094 | 10.265 | **0.001*** | 0.055 | 3.641 | 0.056 | -0.017 | 0.690 | 0.406 | |
| Obsessive-compulsive | 70 | 0.001 | 0.001 | 0.975 | 0.080 | 7.423 | **0.006*** | -0.024 | 1.657 | 0.198 | |
| Interpersonal sensibility | 70 | 0.033 | 1.281 | 0.258 | 0.058 | 3.315 | 0.069 | -0.024 | 1.748 | 0.186 | |
| Depression | 70 | 0.067 | 1.998 | 0.157 | 0.054 | 3.047 | 0.081 | -0.022 | 1.432 | 0.231 | |
| Anxiety | 70 | 0.038 | 2.124 | 0.145 | 0.059 | 4.106 | **0.043** | -0.023 | 1.591 | 0.207 | |
| Anger-hostility | 70 | 0.085 | 9.024 | **0.003*** | 0.063 | 5.123 | **0.024*** | -0.045 | 4.135 | **0.042*** | |
| Phobic-anxiety | 70 | 0.038 | 2.766 | 0.096 | 0.065 | 5.644 | **0.018*** | -0.023 | 1.430 | 0.232 | |
| Paranoid ideation | 70 | 0.047 | 4.548 | **0.033*** | 0.062 | 5.328 | **0.021*** | -0.032 | 2.626 | 0.105 | |
| Psychoticism | 70 | 0.072 | 7.147 | **0.008*** | 0.051 | 3.143 | 0.076 | -0.021 | 1.107 | 0.293 | |
| Processing time | 64 | -0.002 | 1.760 | 0.185 | 0.088 | 9.143 | **0.002*** | -0.034 | 2.021 | 0.155 | |
| The table shows the valid number of subjects analyzed (N) and the results of binary logistic regression analyses. Median split of MDRS-22 scores used to separate patients into HMD and LMD subgroups: ≥1.70 for men, ≥1.82 for women. SCL-90-R Symptom Checklist-90-Revised, BDI-II Beck Depression Inventory-II, GSI Global Severity Index, PST Positive Symptom Total, PSDI Positive Symptom Distress Index. P < 0.05 in bold, * significant in bootstrap analysis. Coding: Patients with low masculine depression scores = 0 vs. Patients with high masculine depression scores = 1. | | | | | | | | | | |  |

| **Supplementary Table S5** Binary logistic regression to differentiate between patients with high masculine depression scores and healthy control subjects using the SCL-90-R values as primary predictor adjusted for sex and age | | | | | | | | | | |  |  |
| --- | --- | --- | --- | --- | --- | --- | --- | --- | --- | --- | --- | --- |
|  |  | Primary predictor | | | Sex | | | Age | | | |  |
|  | N | B | Wald | P | B | Wald | P | B | Wald | P | |  |
| **Dependent Variable: Patients with high masculine depression scores vs. healthy control subjects** |  |  |  |  |  |  |  |  |  |  | |  |
| **Primary predictor: SCL-90-R values** |  |  |  |  |  |  |  |  |  |  | |  |
| GSI | 255 | 0.369 | 24.308 | **< 0.001*** | 1.472 | 1.430 | 0.232 | -0.012 | 0.143 | 0.706 | |  |
| PSDI | 255 | 0.312 | 50.904 | **< 0.001*** | -0.298 | 0.207 | 0.649 | 0.035 | 2.447 | 0.118 | |  |
| PST | 255 | 0.376 | 32.988 | **< 0.001*** | 1.615 | 3.689 | 0.055* | -0.005 | 0.038 | 0.845 | |  |
| Somatization | 255 | 0.279 | 52.236 | **< 0.001*** | -0.013 | 0.001 | 0.981 | 0.013 | 0.506 | 0.477 | |  |
| Obsessive-compulsive | 255 | 0.286 | 45.017 | **< 0.001*** | 1.382 | 2.932 | 0.087 | -0.017 | 0.500 | 0.480 | |  |
| Interpersonal sensibility | 255 | 0.255 | 51.765 | **< 0.001*** | 1.063 | 2.742 | 0.098 | -0.013 | 0.479 | 0.489 | |  |
| Depression | 255 | 0.414 | 18.435 | **< 0.001*** | 2.672 | 2.979 | 0.084* | 0.034 | 0.495 | 0.482 | |  |
| Anxiety | 255 | 0.244 | 56.802 | **< 0.001*** | 0.291 | 0.219 | 0.640 | 0.006 | 0.078 | 0.781 | |  |
| Anger-hostility | 255 | 0.287 | 59.534 | **< 0.001*** | 0.102 | 0.037 | 0.847 | -0.007 | 0.114 | 0.735 | |  |
| Phobic-anxiety | 255 | 0.393 | 41.129 | **< 0.001*** | 0.519 | 0.629 | 0.428 | -0.001 | 0.003 | 0.955 | |  |
| Paranoid ideation | 255 | 0.229 | 61.148 | **< 0.001*** | 0.721 | 2.196 | 0.138 | -0.016 | 0.954 | 0.329 | |  |
| Psychoticism | 255 | 0.370 | 39.428 | **< 0.001*** | 0.973 | 1.670 | 0.196 | 0.009 | 0.180 | 0.671 | |  |
| Processing time | 230 | 0.012 | 48.457 | **< 0.001*** | 0.315 | 0.770 | 0.380 | -0.027 | 3.453 | 0.063 | |  |
| The table shows the valid number of subjects analyzed (N) and the results of binary logistic regression analyses. Median split of MDRS-22 scores used to separate patients into HMD and LMD subgroups: ≥1.70 for men, ≥1.82 for women. SCL-90-R Symptom Checklist-90-Revised, GSI Global Severity Index, PST Positive Symptom Total, PSDI Positive Symptom Distress Index. P < 0.05 in bold, *also significant in bootstrap analysis. Coding: healthy control subjects = 0 vs. Patients with high masculine depression scores = 1; Females = 0 vs. Males = 1. | | | | | | | | | | |  | |

| **Supplementary Table S6** Binary logistic regression to differentiate between patients with low masculine depression scores and healthy control subjects using the SCL-90-R values as primary predictor adjusted for sex and age | | | | | | | | | | |  |  |
| --- | --- | --- | --- | --- | --- | --- | --- | --- | --- | --- | --- | --- |
|  |  | Primary predictor | | | Sex | | | Age | | | |  |
|  | N | B | Wald | P | B | Wald | P | B | Wald | P | |  |
| **Dependent Variable: Patients with low masculine depression scores vs. healthy control subjects** |  |  |  |  |  |  |  |  |  |  | |  |
| **Primary predictor: SCL-90-R values** |  |  |  |  |  |  |  |  |  |  | |  |
| GSI | 256 | 0.309 | 51.562 | **< 0.001*** | 1.319 | 4.374 | **0.037** | 0.047 | 5.193 | **0.023*** | |  |
| PSDI | 256 | 0.294 | 61.162 | **< 0.001*** | 0.172 | 0.137 | 0.711 | 0.078 | 18.991 | **< 0.001*** | |  |
| PST | 256 | 0.278 | 57.782 | **< 0.001*** | 1.237 | 5.229 | **0.022*** | 0.046 | 6.454 | **0.011*** | |  |
| Somatization | 256 | 0.203 | 54.460 | **< 0.001*** | 0.007 | 0.000 | 0.985 | 0.048 | 13.257 | **< 0.001** | |  |
| Obsessive-compulsive | 256 | 0.235 | 58.268 | **< 0.001*** | 0.914 | 2.417 | 0.120 | 0.036 | 3.509 | 0.061 | |  |
| Interpersonal sensibility | 256 | 0.162 | 62.456 | **< 0.001*** | 0.426 | 1.118 | 0.290 | 0.030 | 5.132 | **0.023*** | |  |
| Depression | 256 | 0.309 | 42.178 | **< 0.001*** | 1.980 | 5.903 | **0.015*** | 0.081 | 8.381 | **0.004*** | |  |
| Anxiety | 256 | 0.210 | 51.436 | **< 0.001*** | 0.447 | 1.071 | 0.301 | 0.039 | 6.820 | **0.009*** | |  |
| Anger-hostility | 256 | 0.213 | 48.449 | **< 0.001*** | 0.126 | 0.126 | 0.723 | 0.035 | 7.959 | **0.005*** | |  |
| Phobic-anxiety | 256 | 0.244 | 36.597 | **< 0.001*** | -0.045 | 0.013 | 0.910 | 0.045 | 10.735 | **0.001*** | |  |
| Paranoid ideation | 256 | 0.133 | 35.673 | **< 0.001*** | 0.092 | 0.080 | 0.778 | 0.026 | 5.432 | **0.020*** | |  |
| Psychoticism | 256 | 0.270 | 51.037 | **< 0.001*** | 0.516 | 1.569 | 0.210 | 0.036 | 6.145 | **0.013*** | |  |
| Processing time | 234 | 0.010 | 42.428 | **< 0.001*** | 0.119 | 0.107 | 0.744 | 0.029 | 4.886 | **0.027*** | |  |
| The table shows the valid number of subjects analyzed (N) and the results of binary logistic regression analyses. Median split of MDRS-22 scores used to separate patients into HMD and LMD subgroups: ≥1.70 for men, ≥1.82 for women. SCL-90-R Symptom Checklist-90-Revised, GSI Global Severity Index, PST Positive Symptom Total, PSDI Positive Symptom Distress Index. P < 0.05 in bold, *also significant in bootstrap analysis. Coding: healthy control subjects = 0 vs. Patients with low masculine depression scores = 1; Males = 1 vs. Females = 2. | | | | | | | | | | |  | |

1. Rice, S.M., et al., *Development and preliminary validation of the male depression risk scale: furthering the assessment of depression in men.* J Affect Disord, 2013. **151**(3): p. 950–8.

2. von Zimmermann, C., et al., *Masculine depression and its problem behaviors: use alcohol and drugs, work hard, and avoid psychiatry!* Eur Arch Psychiatry Clin Neurosci, 2023. **274**(2): p. 321–333.
